# Everolimus-Induced Pulmonary Toxicity in a Lung Transplant Recipient: A Case Report

**DOI:** 10.7759/cureus.95593

**Published:** 2025-10-28

**Authors:** Fabio Varón-Vega, Maria C Martinez-Ayala, Camilo Rodríguez, David Mendoza, Luis J Tellez, Eduardo Tuta-Quintero

**Affiliations:** 1 Critical Care and Lung Transplantation Service, Fundación Neumológica Colombiana, Bogotá, COL; 2 Epidemiology and Biostatistics, Fundación Cardioinfantil-Instituto de Cardiología, Bogotá, COL; 3 Pulmonology, Fundación Neumológica Colombiana, Bogotá, COL; 4 Pulmonary and Critical Care Medicine, Fundación Neumológica Colombiana, Bogotá, COL; 5 Thoracic Surgery, Fundación Cardioinfantil - Instituto de Cardiología, Bogotá, COL; 6 Medicine, Universidad de La Sabana, Chía, COL

**Keywords:** everolimus, graft rejection, immunosuppression therapy, lung transplantation, organizing pneumonia

## Abstract

Drug-induced pneumonitis is a recognized yet often underdiagnosed complication of mammalian target of rapamycin (mTOR) inhibitors - immunosuppressive agents such as everolimus that block cellular proliferation pathways - particularly among solid organ transplant recipients. Diagnosis is frequently delayed because clinical, radiologic, and histopathologic findings can overlap with infectious or autoimmune diseases. We describe a 66-year-old man who developed progressive exertional dyspnea and facial rash six months after initiation of everolimus and 18 months after undergoing bilateral lung transplantation. He required supplemental oxygen and was admitted to a step-down respiratory care unit. Extensive infectious and autoimmune workups were negative. Lung biopsy revealed acute fibrinous and organizing pneumonia (AFOP). Everolimus was discontinued, and his immunosuppression was switched back to low-dose tacrolimus plus prednisone, leading to rapid clinical improvement and complete radiologic resolution within four weeks. This case underscores the need for high clinical suspicion of everolimus-induced pneumonitis in transplant recipients presenting with new pulmonary infiltrates and highlights the importance of early drug discontinuation guided by histopathologic confirmation to prevent progression to respiratory failure.

## Introduction

Lung transplantation is a life-saving therapy for patients with advanced pulmonary diseases, but its long-term success is frequently limited by complications such as acute rejection, chronic graft dysfunction, and immunosuppressive drug toxicities [1,2]. The diagnostic process is particularly challenging because these entities often share overlapping clinical, radiologic, and histopathologic features [2].

Standard post-transplant immunosuppressive regimens typically include calcineurin inhibitors (CNIs), antimetabolites, and corticosteroids [3]. However, alternative agents may be required when intolerance or adverse effects occur. Among these, mammalian target of rapamycin (mTOR) inhibitors - such as everolimus - are increasingly used in lung transplant recipients, particularly for CNI minimization or renal preservation, with reported use in up to 15-25% of programs worldwide [3,4]. Although effective, mTOR inhibitors have been associated with non-infectious interstitial pneumonitis (NIP), a potentially serious yet underrecognized complication [5]. Its incidence is estimated at approximately 0.4% in transplant populations and up to 30% in oncologic settings [6,7].

The clinical presentation of mTOR-associated NIP is heterogeneous, and its underlying mechanisms remain incompletely understood, with proposed explanations including direct alveolar toxicity and immune-mediated injury [8]. Distinguishing NIP from acute rejection is particularly difficult due to overlapping manifestations, underscoring the need for a multidisciplinary diagnostic approach incorporating histopathology, bronchoalveolar lavage, and rigorous exclusion of infection [5,8]. We report a lung transplant recipient with prior intolerance to multiple immunosuppressive agents who developed a complex respiratory and systemic syndrome shortly after everolimus initiation.

## Case presentation

A 66-year-old man, 18 months after bilateral lung transplantation for interstitial pneumonitis, was receiving immunosuppressive therapy with everolimus (0.75 mg twice daily, most recent trough level: 5.2 ng/mL), azathioprine (100 mg/day), and prednisone (10 mg/day). He had a history of intolerance to tacrolimus (nephrotoxicity) and mycophenolate mofetil (hematologic toxicity).

**Figure 1:**
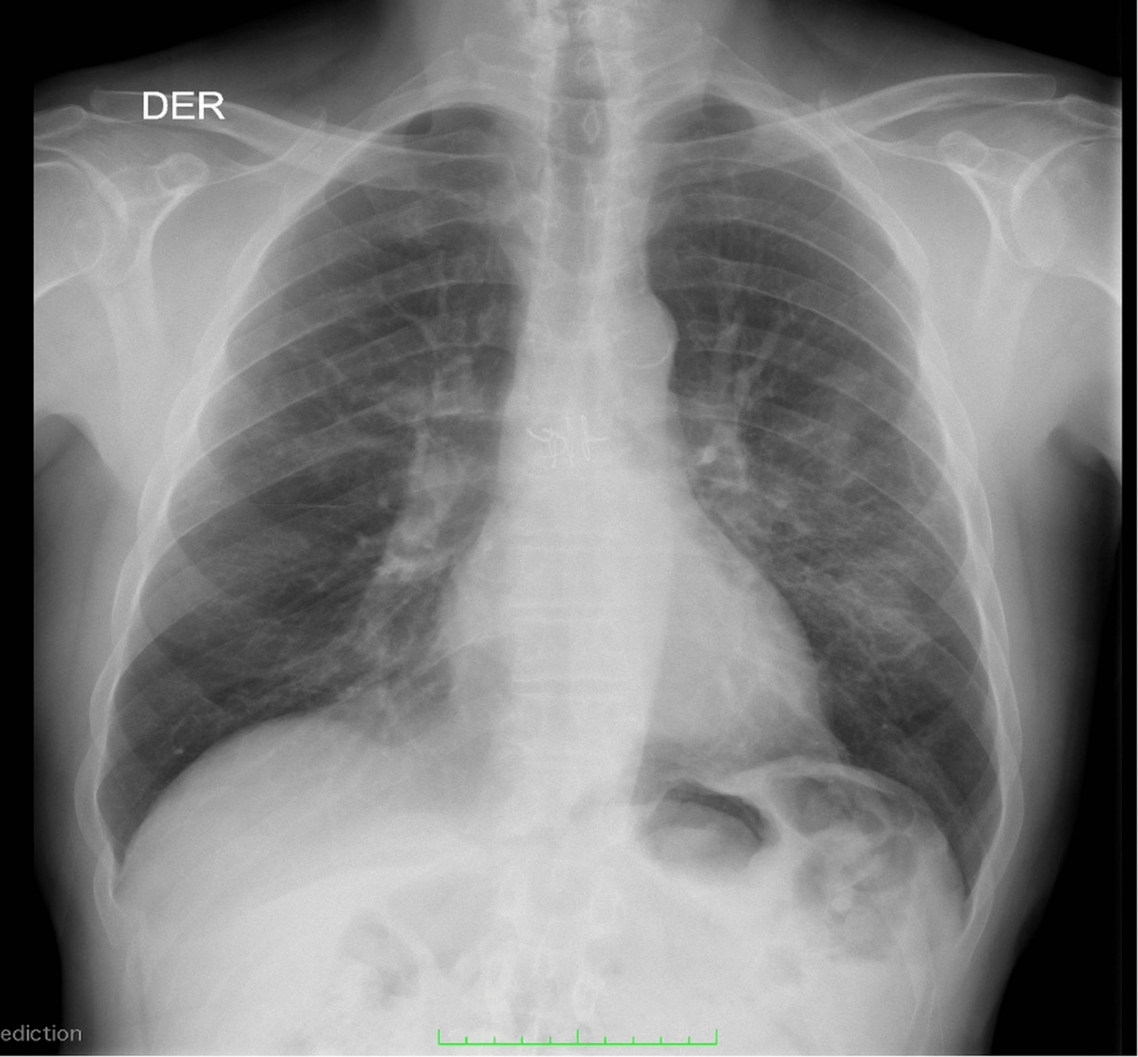
Admission chest radiograph

**Figure 2:**
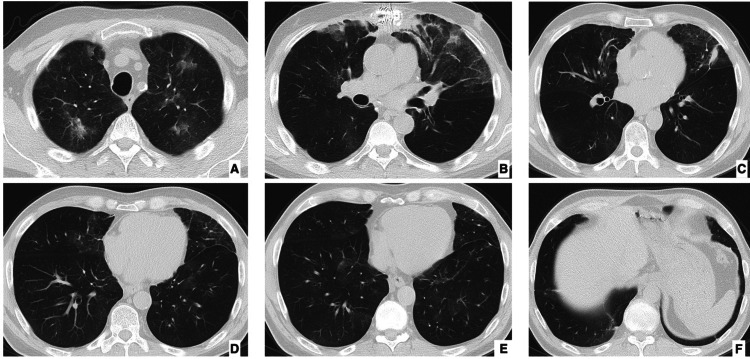
Admission chest computer tomography Axial chest CT images at multiple levels (A–F) showing patchy ground-glass opacities and consolidations with a peripheral and posterior predominance, associated with interlobular septal thickening and vascular enlargement.

Two weeks before presentation, he developed progressive exertional dyspnea and nonproductive cough, followed by oxygen desaturation to 85%, periorbital edema, frontal and malar facial rash, and persistent chest pain and headache. The rash showed partial transient improvement after outpatient prednisone was empirically increased to 20 mg/day. Outpatient evaluation had revealed new bilateral pulmonary infiltrates unresponsive to broad-spectrum antimicrobial therapy. At presentation, a chest radiograph was obtained, followed by high-resolution computed tomography, which showed bilateral apical ground-glass opacities and lingular septal thickening, more prominent on the left side (Figures 1-2). 

A comprehensive infectious workup, including bronchoscopy with bronchoalveolar lavage (BAL), respiratory pathogen panel, and mycobacterial polymerase chain reaction from both BAL fluid and tissue, was negative. Cytomegalovirus viral load was undetectable (<1.53 log IU/mL). Autoimmune serologies were also repeated and unremarkable: serum immunoglobulin G was within normal limits (>3.3 g/L); antinuclear antibodies, anticentromere, anti-cyclic citrullinated peptide, and antineutrophil cytoplasmic antibodies were negative; anti-ribonucleoprotein, anti-Ro/SSA, anti-Smith, anti-histidyl-tRNA synthetase, and anti-topoisomerase I were below threshold levels; and complement levels were preserved complement component 3 (C3) of 1.08 g/L and complement component 4 (C4) of 0.23 g/L. Pulmonary function tests, obtained one week prior, showed a new decline in diffusing capacity of the lung for carbon monoxide to 42% predicted. Given the persistence of symptoms and lack of response to empiric therapy, a lung biopsy was performed five days after admission.

**Figure 3:**
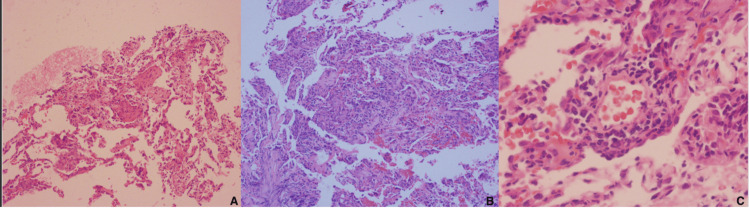
Lung biopsy Lung biopsy showing acute lung injury with a pattern of acute fibrinous and organizing pneumonia (AFOP) and features consistent with severe acute cellular rejection (A4B1R). Panels A–C illustrate different sections at increasing magnification.

Histopathological analysis (Figure 3) revealed features of acute fibrinous and organizing pneumonia (AFOP), a rare and nonspecific histological pattern of lung injury associated with infections, autoimmune diseases, and drug toxicity. In this case, the presence of cutaneous manifestations, progressive respiratory deterioration, ongoing exposure to everolimus, and the exclusion of infectious and autoimmune etiologies strongly supported the diagnosis of everolimus-induced pneumonitis.

**Figure 4:**
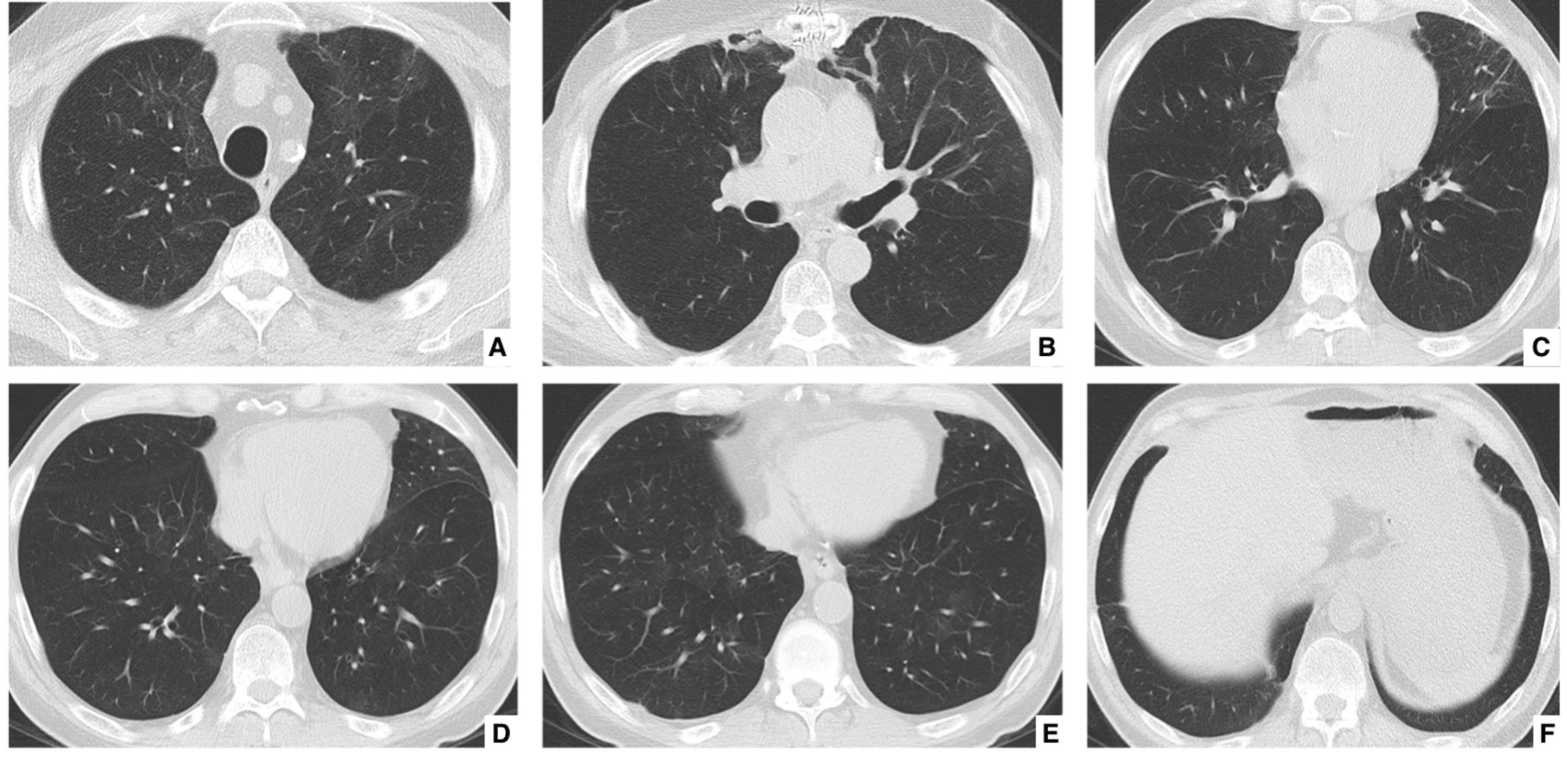
Post-everolimus discontinuation follow-up Follow-up axial chest CT images at the same anatomical levels (A–F) demonstrating resolution of the previously seen ground-glass opacities and consolidations.

Given the working diagnosis of drug-induced lung toxicity, everolimus was discontinued. High-dose intravenous methylprednisolone was initiated (500 mg/day for three days, followed by oral taper). In light of prior nephrotoxicity but the need for continued immunosuppression, tacrolimus was cautiously reintroduced with gradual titration targeting trough levels of 4-6 ng/mL with close renal monitoring; serum creatinine remained stable (1.2-1.4 mg/dL). Azathioprine was continued. Following this intervention, the patient experienced marked clinical improvement, with resolution of the facial rash, stabilization of respiratory symptoms, and radiological improvement on follow-up imaging at four weeks (Figure 4). Follow-up duration now exceeds three months without recurrence of symptoms.

## Discussion

Everolimus is an mTOR inhibitor increasingly used in lung transplant recipients, particularly in patients with intolerance to CNIs due to nephrotoxicity or neurotoxicity. Although effective in reducing chronic allograft dysfunction and offering a lower risk of nephrotoxicity, everolimus is associated with a variety of adverse effects, including hematologic, metabolic, and pulmonary toxicities. Among these, NIP is a rare but potentially serious complication that may go unrecognized without a high index of suspicion [4-7]. Early clinical “red flags” that should prompt suspicion include new or worsening exertional dyspnea, dry cough without infectious prodrome, low-grade or unexplained fever, hypoxemia disproportionate to imaging findings, and, as in this case, concurrent cutaneous eruptions.

The reported incidence of mTOR inhibitor-induced pneumonitis varies by population. In transplant recipients, it is estimated at 0.4%, while, in oncology settings, particularly among patients receiving everolimus for renal cell carcinoma or breast cancer, it can reach up to 30% [5,6]. The underlying mechanisms remain incompletely understood. Proposed pathophysiologic pathways include direct toxicity to alveolar epithelial cells, immune-mediated responses such as lymphocytic alveolitis, and cytokine dysregulation leading to alveolar-capillary injury [9,8]. The latency period between drug initiation and symptom onset is highly variable, ranging from weeks to several months, further complicating timely recognition.

Clinically, patients with everolimus-induced pneumonitis may present with nonspecific symptoms, such as cough, progressive dyspnea, low-grade fever, hypoxemia, and fatigue. Extrapulmonary findings, such as cutaneous rashes, as seen in this case, are rarely reported and remain poorly characterized in the literature, representing one of this report’s unique contributions. Radiologic features are similarly nonspecific, often demonstrating bilateral ground-glass opacities, septal thickening, and consolidations, typically with a peripheral or upper-lobe predominance [10,11]. However, these imaging patterns overlap with infections, autoimmune diseases, and other forms of drug toxicity, making histologic confirmation essential in uncertain cases.

The presence of AFOP in the lung biopsy was a decisive finding in this case. AFOP is a rare histologic pattern of lung injury first described by Beasley et al. and characterized by intra-alveolar fibrin deposition (“fibrin balls”) and organizing pneumonia without prominent hyaline membranes [9]. It has been associated with various etiologies, including infections, autoimmune diseases, drug reactions, and even idiopathic presentations. Importantly, AFOP has been reported in association with mTOR inhibitor toxicity, supporting the causal link in this case [12,13]. This case adds to the limited literature by presenting the unusual coexistence of AFOP with concurrent cutaneous involvement.

In terms of management, the cornerstone of treatment is prompt discontinuation of the offending agent; however, clinicians must carefully balance the risk of acute allograft rejection against the risk of progressive lung injury. In this patient, everolimus was withdrawn, and tacrolimus was cautiously reintroduced, with strict monitoring of renal function and drug levels. Corticosteroid therapy remains the standard of care, particularly when initiated early, as was done with methylprednisolone pulses in this case. It is also critical to exclude opportunistic infections - such as fungal pathogens, cytomegalovirus, *Pneumocystis jirovecii*, and atypical mycobacteria - before attributing findings solely to drug toxicity [14].

This case underscores the diagnostic complexity of pulmonary complications in lung transplant recipients, especially in the setting of sequential immunosuppression with overlapping toxicities. Its relevance extends beyond lung transplantation, given that mTOR inhibitors are increasingly used in renal, liver, and heart transplant populations. The recognition of AFOP with cutaneous involvement as a possible manifestation of everolimus toxicity emphasizes the need for heightened clinical vigilance and multidisciplinary evaluation. Further research is needed to better define dose-toxicity thresholds, risk stratification strategies, and optimal monitoring protocols to guide safer immunosuppressive management.

## Conclusions

Everolimus-induced pneumonitis is a rare but clinically significant complication in lung transplant recipients and should be suspected in the presence of unexplained respiratory symptoms or cutaneous manifestations after exclusion of infectious and autoimmune causes. Histopathologic confirmation, including AFOP when present, supports diagnostic certainty. Timely recognition, withdrawal of everolimus, and early corticosteroid therapy are crucial for reversal, as demonstrated in this case. Given the expanding use of mTOR inhibitors, clinician awareness and a multidisciplinary approach remain vital to ensure prompt diagnosis and optimal management.
